# Living Conditions and* Helicobacter pylori* in Adults

**DOI:** 10.1155/2017/9082716

**Published:** 2017-10-12

**Authors:** Odete Amaral, Isabel Fernandes, Nélio Veiga, Carlos Pereira, Claudia Chaves, Paula Nelas, Daniel Silva

**Affiliations:** ^1^CI&DETS, Polytechnic Institute of Viseu, Viseu, Portugal; ^2^Health Sciences Institute, Universidade Católica Portuguesa, Lisbon, Portugal; ^3^Centre for Interdisciplinary Research in Health (CIIS), Universidade Católica Portuguesa, Lisbon, Portugal

## Abstract

**Introduction:**

Infection by the bacterium* Helicobacter pylori* (*H. pylori*) is transmissible and is considered a public health issue which affects people of all ages. The objective of this study was to identify factors (lifestyles, dietary factors, and hygiene conditions) related to the prevalence of* H. pylori* infection.

**Methods:**

We carried out an observational cross-sectional study with a community sample of adults from the municipalities of Viseu and Sátão, Portugal. The final sample resulted in 166 adults. The data were collected through a self-administered questionnaire with questions regarding sociodemographic aspects and lifestyles.* H. pylori* infection was identified using the 13C-urea breath test.

**Results:**

No association was found between the prevalence of* H. pylori* infection and the use of tobacco, alcohol, or coffee or dietary factors. The prevalence of* H. pylori* infection was higher in adults who reported higher consumption of fried food and lower consumption of vegetables and fruit.* H. pylori* infection was significant for the variables of lower frequency of handwashing before going to the bathroom (*p* = 0.02) and well water consumption (*p* = 0.05).

**Conclusion:**

A significant association was found for* H. pylori* infection with the lower frequency of handwashing before going to the bathroom and the consumption of well water.

## 1. Introduction

*H. pylori* is considered to be a chronic and transmissible infection, even though the exact chain of transmission is not completely known. It is believed that human beings are practically the only natural reservoir of* H. pylori* [1]. Research suggests that contact with the bacterium occurs predominantly during childhood and between direct family members (intrafamilial transmission) [2, 3]. Intrafamilial transmission appears to be the main route for the acquisition of this infection, especially among mothers and children and among siblings, supporting the hypothesis that close contact is crucial for the transmission of the infection [4, 5]. In developing countries, studies report that the hygiene conditions and the surrounding environment influence the transmission of* H. pylori* [6, 7]. Person-to-person transmission through oral-to-oral or fecal-to-oral routes is considered the most probable, and this pathogen can be transmitted orally through fecal matter by the ingestion of water contaminated with waste [1]. Risk factors related to* H. pylori* infection include poor socioeconomic status, poor hygiene, inadequate sanitation conditions, overcrowding, consumption of contaminated water and food, and bacterial infection within the household [1, 8]. The improvement of hygiene standards, mainly due to the implementation of basic sanitation, a decrease in the number of close contacts, and, possibly, an increase in the consumption of antibiotics, contributed to a gradual variation in the frequency of infection in the different phases of the life cycle. In other words, this infection now has a higher incidence in the later stages of childhood, adolescence, and adulthood [4, 9]. Therefore, the identification of the determinants of* H. pylori* infection in different phases of the life cycle is essential to understand its increasing incidence in certain countries and populations, development, and health consequences in the human body.

A study from Brazil reported an increase in* H. pylori* infection associated with a higher number of siblings, schooling since nursery school, and housing with poor conditions and no paved roads, which can be considered important indicators associated with poor living conditions [10]. Poor sanitation conditions and overcrowding may also constitute important risk factors for* H. pylori* infection. Likewise, the number of people per room and the number of children in a household were also identified as independent risk factors for* H. pylori* infection [11]. Another study conducted in Germany indicated a positive association for* H. pylori* infection with “more than three children living in the household” (OR = 2.4; *p* = 0.001), “more people living per square meter above the average” (OR = 1.4; *p* = 0.03), “the house being located on the main road” (OR = 1.4; *p* = 0.04), and “consumption of well water” (OR = 2.3; *p* = 0.05) [12]. However, the functional components of many other associated factors are not fully clarified.

Accordingly, the objective of this study was to identify factors (lifestyles, dietary factors, and hygiene conditions) related to the prevalence of* H. pylori* infection.

## 2. Participants and Methods

We carried out an observational epidemiological cross-sectional study. The sample consisted of 166 adult individuals from the municipalities of Viseu and Sátão, Portugal. The majority of the sample were female (56.6%) with a mean age of 46.96 ± 3.17 years (minimum of 19 years old and maximum of 92 years old). The majority of the sample were aged ≤50 years (54.8%), were married or lived in a nonmarital relationship (64.5%), held a bachelor's or a licentiate's degree (35.2%), and were employed (70.9%). The exclusion criteria defined for the present study were as follows: the participant was medicated with antibiotics in the past 30 days, ate any kind of food or drank beverages in the last 6 hours, and underwent any kind of eradication treatment before or at the time of the 13C-urea breath test, in order to avoid the presence of fake negatives in the present study.

We collected the data through a self-administered questionnaire divided into three main sections: (I) sociodemographic variables (gender, age, marital status, residence area, scholarship, professional situation, and crowding index) with a total of 10 questions; (II) clinical situation (diseases and other health outcomes and daily medication) with a total of 6 questions; (III) household composition and lifestyles (dietary factors, tobacco and coffee consumption, physical exercise, hygiene practices, and household conditions) with a total of 12 questions.

In order to assess the presence of* H. pylori*, we used the 13C-urea breath test, which is considered a noninvasive test and of easy application in community observational epidemiological studies. The test was performed in the morning at the home of the participant and in the presence of the main researcher, after at least 6 hours of fasting by the participant. The protocol consisted in the exhalation of carbon dioxide in samples before and after swallowing urea labeled with nonradioactive carbon-13. The samples were then analyzed and each result would be classified as positive or negative for* H. pylori* infection.

The study was submitted and approved by the Ethics Committee of the Education School of Health of the Polytechnic Institute of Viseu, Portugal. The data collection instrument was voluntarily answered by adults, and the confidentiality and anonymity of the information collected were guaranteed.

After collecting the data, the questionnaires were numbered, stored, and processed, using the Statistical Package for the Social Sciences (SPSS version 23.0). The prevalence was expressed in proportions and the odds ratio (OR) with a 95% confidence interval (CI) was used to measure the strength of the association between variables. Proportions were compared by the chi-square test. The level of significance was set at 5% (*p* < 0.05).

## 3. Results

The prevalence of* H. pylori* was 48.8%, higher in females (50.0% versus 47.2%, *p* = 0.72), in individuals over 40 years of age (51.8% versus 42.9, *p* = 0.27), in individuals with lower academic qualifications (≤12th grade 53.3% versus >12th grade 43.8%, *p* = 0.23), and in adults who reported having two or more siblings (53.8% versus 42.7%, *p* = 0.15), but with no statistical differences for the sociodemographic variables.

Regarding the lifestyle and the presence of* H. pylori*, Table 1 shows that there was no association between the prevalence of* H. pylori* infection and the use of tobacco, alcohol, or coffee. The prevalence of* H. pylori* infection is higher in individuals who do not drink alcohol, do not smoke, and do not drink coffee. Nevertheless, no statistically significant association was found. Also, among adults who drink coffee, the prevalence for* H. pylori* infection is higher in those who report drinking 2 or more coffees a day, without any significant differences.

The results in Table 2 suggest that the* H. pylori* infection was higher for individuals who reported that they had never eaten or would rarely eat fried food, when compared to adults who reported eating fried food at times or almost every day (OR = 1.04, 95% CI: 0.56–1.92). It increased for individuals who ate vegetables less frequently (50.0%) in comparison to those who ate them almost every day or every single day (48.8%, *p* = 0.84). In contrast, the prevalence of* H. pylori* was higher in people who did not drink milk (55.2% versus 47.4%; *p* = 0.45) and in adults who reported drinking soft drinks (OR = 1.33, 95% CI: 0.71–2.49). However, we did not find any dietary factor with a significant association with* H. pylori* infection.

Most people reported having piped water (*n* = 162; 97.6%), but 4 people (2.4%) reported not having piped water. In relation to the sewage system, most of the sample reported that they have a sewage network linked to their household (*n* = 159, 95.8% versus *n* = 7, 4.2%).

When we analyzed the relation between the prevalence of* H. pylori* infection and hygiene conditions (Table 3), we found that individuals who mentioned having washed their hands before going to the bathroom rarely or sometimes had a lower risk than those who had never done this (OR = 0.40, 95% CI: 0.18–0.87). Also, the consumption of well water is positively related to* H. pylori* infection (OR = 2.13, 95% CI: 1.00–4.64), although the statistical association is marginal. In relation to all the other variables analyzed, we did not find any statistically significant differences.

## 4. Discussion

In the current study, we intend to identify dietary factors, lifestyles, and hygiene conditions associated with* H. pylori* infection. We did not find any significant differences between* H. pylori* infection and coffee, alcohol, and tobacco consumption. Evidence is also not consensual about the association with these variables [13]. A study carried out in Japan showed that smoking was negatively related to* H. pylori* infection [14]. Some studies show a positive relationship while others have found no relationship between smoking and* H. pylori* infection [1, 13, 14]. Other studies have shown an association between* H. pylori* infection and tobacco consumption and the number of cigarettes per day (suggesting that the risk of* H. pylori* infection decreased with the daily cigarette consumption) [1]. Regarding alcohol consumption, previous studies have found a relationship between* H. pylori* infection and alcohol consumption, although most of them did not find a significant association [15, 16]. Interestingly, in a cross-sectional study of 447 adults with a positivity evaluation of* H. pylori* using the 13C-urea breath test, Brenner found a 21% prevalence of infection and suggested a negative dose-response relation with alcohol consumption (consumption of > 75 g ethanol/week after adjusting the variables for gender, age, educational level, nationality, and family history of ulcer; OR = 0.33, 95% CI: 0.16–0.68) and a positive dose-response association with coffee consumption (less than 3 coffees; OR = 1.49, 95% CI: 0.71–3.12; ≥3 coffees; OR = 2.49, 95% CI: 1.23–5.03) [17].

Regarding hygiene conditions, studies have shown an increase in* H. pylori* infection associated with a greater number of siblings and housing in a street with unpaved roads and no sanitary conditions, indicating worse living conditions, the use of well water, and a higher agglomeration index as risk factors [10, 12]. In this study, we observed a borderline association with well water consumption (*p* = 0.05) and an association with washing hands before going to the bathroom (*p* = 0.02).

With regard to dietary factors and the infection, studies have reported that some dietary risks for gastritis are also considered as risk factors for* H. pylori* [18, 19].

Once again, there is evidence that* H. pylori* is associated with the consumption of contaminated water, but not food. It is thought that person-to-person contact is the most likely mode of transmission, and there is no direct evidence that food is involved in the transmission of* H. pylori* [20]. This association between food and* H. pylori* infection will be related not only to the type of diet (healthy, unhealthy), but also especially to the consumption of contaminated food, in which this contamination will be higher when consuming contaminated raw vegetables and fruit. In developing countries, the consumption of drinking water and vegetable products contaminated by sewage can be a serious risk [21]. The consumption of vegetables and fruit, raw and contaminated, (fertilized with feces), was considered a risk factor for infection as well as the consumption of contaminated drinking water [21–23].

## 5. Conclusion

Among the food factors, lifestyles, and hygiene conditions, we found a significant association for* H. pylori* infection with the lower frequency in handwashing before going to the bathroom and the consumption of well water. Additional research should address the relations observed in representative population samples and aim to gain an understanding of their underlying mechanisms. Thus, the identification of the determinants of* H. pylori* infection in different phases of the life cycle is essential to understand the association with the different outcomes that can be caused by the infection and, therefore, understand better the influence that it may have in different populations.

## Figures and Tables

**Table 1:** Relationships between the prevalence of *H. pylori* infection and lifestyles.

	*H. pylori* positive	*H. pylori* negative	Total	Prevalence of *H. pylori* (%)	OR (95% CI)	*p*
Alcohol consumption						
Yes	22	31	53	41.5	0.65 (0.34–1.26)	0.20
No	59	54	113	52.2	1^*∗*^	
Tobacco consumption						
Yes	20	31	51	39.2	0.57 (0.29–1.12)	0.10
No	61	54	115	53.0	1^*∗*^	
Number of cigarettes per day						
≤10 cigarettes	14	19	33	42.4	1.33 (0.36–4.83)	0.67
>10 cigarettes	5	9	14	35.7	1^*∗*^	
Coffee consumption						
Yes	56	63	119	47.1	0.75 (0.40–1.54)	0.48
No	25	22	47	53.2	1^*∗*^	
Number of coffees per day						
1	26	36	62	41.9	0.61 (0.30–1.30)	0.21
2 or more	28	24	52	53.8	1^*∗*^	

^*∗*^Reference group.

**Table 2:** Association between the prevalence of *H. pylori* infection and dietary factors.

	*H. pylori* positive	*H. pylori* negative	Total	Prevalence of *H. pylori* (%)	OR (95% CI)	*p*
Frequency of eating fried food						
Never/rarely	36	37	73	49.3	1.04 (0.56–1.92)	0.91
Sometimes/almost every day	45	48	93	48.4	1^*∗*^	
Frequency of eating vegetables						
Rarely/sometimes	25	25	50	50.0	1.07 (0.55–2.08)	0.84
Almost every day/every day	56	60	116	48.3	1^*∗*^	
Frequency of eating fruit						
Never/sometimes	18	19	37	48.6	0.99 (0.48–2.06)	0.98
Almost every day/every day	63	66	129	48.8	1^*∗*^	
Consumption of milk						
Yes	65	72	137	47.4	0.73 (0.33–1.64)	0.45
No	16	13	29	55.2	1^*∗*^	
Consumption of soft drinks						
Yes	53	50	103	51.5	1.33 (0.71–2.49)	0.38
No	28	35	63	44.4	1^*∗*^	

^*∗*^Reference group.

**Table 3:** Prevalence of *H. pylori* infection and hygiene conditions (*p* < 0.05 represents statistically significant *p* values obtained in the present study).

	*H. pylori* positive	*H. pylori* negative	Total	Prevalence of *H. pylori* (%)	OR (95% CI)	*p*
Frequency of washing hands before going to the bathroom						
Never	25	16	41	61.0	1^*∗*^	
Rarely/sometimes	30	48	78	38.5	0.40 (0.18–0.87)	**0.02**
Almost always/always	26	21	47	55.3	0.79 (0.34–1.86)	0.59
Frequency of washing hands after going to the bathroom						
Never	6	9	15	40.0	1^*∗*^	
Rarely/sometimes	17	18	35	48.6	1.42 (0.42–4.83)	0.56
Almost always/always	58	58	116	50.0	1.50 (0.50–4.49)	0.47
Frequency of washing hands before meals						
Never	7	8	15	46.7	1^*∗*^	
Rarely/sometimes	38	34	72	52.8	1.28 (0.42–3.90)	0.66
Almost always/always	36	43	79	45.6	0.96 (0.32–2.89)	0.94
Frequency of washing hands before going to sleep						
Never	13	11	24	54.2	1^*∗*^	
Rarely/sometimes	27	33	60	45.0	0.69 (0.27–1.79)	0.45
Almost always/always	41	41	82	50.0	0.85 (0.34–2.11)	0.72
Has ever bitten their nails						
Yes	28	25	53	51.5	1.27 (0.66–2.44)	0.48
No	53	60	113	44.4	1^*∗*^	
Drinking water						
Public network	24	33	57	42.1	0.91 (0.44–1.87)	0.79
Well	29	17	46	63.0	2.13 (1.00–4.64)	**0.05**
Bottled	28	35	63	44.4	1^*∗*^	

^*∗*^Reference group.
